# Single-cell RNA-sequencing reveals the dynamic process and novel markers in porcine spermatogenesis

**DOI:** 10.1186/s40104-021-00638-3

**Published:** 2021-12-07

**Authors:** Lingkai Zhang, Fuyuan Li, Peipei Lei, Ming Guo, Ruifang Liu, Ling Wang, Taiyong Yu, Yinghua Lv, Tao Zhang, Wenxian Zeng, Hongzhao Lu, Yi Zheng

**Affiliations:** 1 Key Laboratory for Animal Genetics, Breeding and Reproduction of Shaanxi Province, College of Animal Science and Technology, Northwest A&F University, Yangling, 712100 Shaanxi China; 2 School of Biological Science and Engineering, Shaanxi University of Technology, Hanzhong, 723001 Shaanxi China; 3 College of Chemistry and Pharmacy, Northwest A&F University, Yangling, 712100 Shaanxi China

**Keywords:** Marker, Pig, scRNA-seq, Spermatogenesis

## Abstract

**Background:**

Spermatogenesis is the process by which male gametes are formed from spermatogonial stem cells and it is essential for the reliable transmission of genetic information between generations. To date, the dynamic transcriptional changes of defined populations of male germ cells in pigs have not been reported.

**Results:**

To characterize the atlas of porcine spermatogenesis, we profiled the transcriptomes of ~ 16,966 testicular cells from a 150-day-old pig testis through single-cell RNA-sequencing (scRNA-seq). The scRNA-seq analysis identified spermatogonia, spermatocytes, spermatids and three somatic cell types in porcine testes. The functional enrichment analysis demonstrated that these cell types played diverse roles in porcine spermatogenesis. The accuracy of the defined porcine germ cell types was further validated by comparing the data from scRNA-seq with those from bulk RNA-seq. Since we delineated four distinct spermatogonial subsets, we further identified CD99 and PODXL2 as novel cell surface markers for undifferentiated and differentiating spermatogonia, respectively.

**Conclusions:**

The present study has for the first time analyzed the transcriptome of male germ cells and somatic cells in porcine testes through scRNA-seq. Four subsets of spermatogonia were identified and two novel cell surface markers were discovered, which would be helpful for studies on spermatogonial differentiation in pigs. The datasets offer valuable information on porcine spermatogenesis, and pave the way for identification of key molecular markers involved in development of male germ cells.

**Supplementary Information:**

The online version contains supplementary material available at 10.1186/s40104-021-00638-3.

## Background

A boar produces several billions of spermatozoa daily [1]. The highly efficient production of spermatozoa relies on spermatogenesis that comprises three stages, i.e., mitosis of spermatogonia, meiosis of spermatocytes, and spermiogenesis (transformation of spherical spermatids) [2]. Mitotic divisions of spermatogonia include self-renewal of spermatogonial stem cells (SSCs) and cell differentiation that gives rise to differentiating type A spermatogonia, intermediate and B spermatogonia. Type B spermatogonia differentiate into spermatocytes that undergo two consecutive rounds of cell divisions and form round spermatids [3, 4]. Subsequently, round spermatids undergo morphological and biochemical changes to form the elongated spermatids that are released into the lumen of seminiferous tubules as spermatozoa [2, 5].

The complicated process of spermatogenesis requires timely coordinated gene expression [6]. Genome-wide microarray and RNA-sequencing (RNA-seq) techniques have been applied to analyze the enriched male germ cell populations or testis samples to obtain the transcriptomes [7–9]. As the process of spermatogenesis is coordinated radially within a cross section of seminiferous tubules and occurs asynchronously along the tubules, there are multiple germ cells at different stages of spermatogenesis, which makes it challenging to obtain stage-specific molecular resolution of germ cell differentiation. As a result, the molecular characterization of spermatogenic cells at a defined stage remains unclear.

A few approaches have been applied to separate the different subtypes of male germ cells. The STA-PUT technique has been used to isolate germ cells in mice, rats, pigs, and cattle, but it usually fails to obtain distinct cell types with high purity [10–14]. The magnetic-activated cell sorting (MACS) and fluorescence-activated cell sorting (FACS) depend on biochemical markers of cells and cell purity is largely affected by the specificity of the chosen antibodies. Moreover, MACS and FACS are unsuitable for separation of spermatocytes or spermatids due to the lack of suitable markers. Recently, the laser capture micro-dissection approach has been used to precisely capture subtypes of male germ cells from paraffin tissue sections, but it leads to RNase contamination after staining [15]. Fortunately, 10 × Genomics single-cell RNA-sequencing (scRNA-seq) is an unbiased approach with high efficiency and sensitivity in cell capture, and it has been used to decipher the dynamic process of spermatogenesis in mouse, primate and sheep testis [16–20]. However, the gene expression profiling of spermatogenic cells at each sub-stage during spermatogenesis and their regulatory networks in pigs have not been uncovered.

Pigs (*Sus scrofa*) are a leading domestic species for meat production. Meanwhile, they are increasingly exploited as an animal model in physiological and pharmacological research [21, 22]. In this study, we profiled ~ 16,966 single-cell transcriptomes from the testis of a 150-day-old pig. This large number of cells allowed us to well identify the populations of germ cells and somatic cells. Using bulk RNA-seq, we provided further supportive evidence on the definition of germ cell clusters. We delineated four distinct spermatogonial subsets in porcine testes and identified novel cell surface markers for undifferentiated and differentiating spermatogonia. The datasheets from the present study could enrich our understanding of spermatogenesis and male reproduction in pigs.

## Methods

### Animals

All experiments utilizing animals were acquired from the farm, Northwest A&F University, Yangling, China. Adult pig testicular tissue was castrated for following experiments. Procured tissue was transported to the laboratory on ice phosphate-buffered saline (PBS; Invitrogen, Carlsbad, CA, USA) with a double dose penicillin/streptomycin (Invitrogen). In all cases, a portion of the testicular tissue was fixed in 4% paraformaldehyde and Bouin’s solution. The bulk of the tissue was used for cell isolation as noted below.

### Generation of cell suspensions

Single-cell suspensions of seminiferous tubules were generated from a 150-day-old Guanzhong pig testis using a two-step enzymatic digestion approach [23]. Briefly, testicular parenchyma was digested with 1 mg/mL Collagenase Type IV (Invitrogen) for 30 min at 34 °C, washed with Dulbecco’s phosphate-buffered saline (DPBS; Invitrogen) to remove interstitial cells, following, the seminiferous tubules were digested with 0.25% trypsin/EDTA (HyClone, Logan, UT, USA) for 5 min at 34 °C, and terminated with 10% FBS. Cell suspensions were obtained by filtering through 40 μm strainers and washed up by DPBS with 0.5 mg/mL DNase I (Sigma, San Francisco, CA, USA) for twice, then suspended in 1640/DMEM (Hyclone) containing 5% FBS.

### Porcine germ cell enrichment by STA-PUT

Cells from adult pig seminiferous tubules were enriched for spermatogonia, spermatocyte or spermatid based on sedimentation velocity at unit gravity [24]. Three adult pigs were used to obtain three independent biological replicates for spermatogonia, spermatocytes and spermatids. Briefly, testis cells suspended in 50 mL buffer plus 0.5% bovine serum albumin (BSA) were loaded onto a 600 mL gradient of 2–4% BSA and allowed to sediment for 3 h at 4 °C. Approximately one hundred 6 mL fractions were collected in plastic tubes and analyzed for content of spermatogonia, spermatocyte or spermatid on the basis of morphology under contrast optics (which typically yields ≥ 80% purity). Fractions containing spermatogonia, spermatocyte or spermatid were pooled separately, concentrated (to ~ 2 × 10^6^ cells/mL) and stored in buffer containing FBS on ice until use.

### Single-cell transcriptomes

Cell suspensions were loaded into Chromium microfluidic chips with 3′ v3 chemistry and used to generate single-cell gel bead emulsions (GEMs) using the Chromium controller (10 × Genomics) per manufacturer recommendations. In all cases, suspensions containing ~ 20,000 cells were loaded on the instrument with the expectation of collecting up to 16,971 GEMs containing single cells. GEM-RT was performed in a T100 Thermal cycler (Bio-Rad) and all subsequent steps to generate single-cell libraries were performed according to manufacturer recommendations. Libraries were sequenced at the Genome Sequencing Facility (GSF) by LC-Bio Technology Co., Ltd (HangZhou, China) on an Illumina NovaSeq 6000 sequencing system. Trimmed FASTQ files (26 bp cell barcode and UMI Read1, and 100 bp Read2), were generated using the CellRanger mkfastq command (a 10 × Genomics wrapper around BCL2Fastq). Primary data analysis (alignment, filtering, and UMI counting) to determine gene transcript counts per cell (producing a gene-barcode matrix) were performed using CellRanger count (10 × Genomics) and Sscrofa11.1 genome assembly/annotation references. All visualization results were completed by R.

### Single-cell RNA-seq analysis

Raw count matrices (10 × Genomics) (Fluidigm C1) were imported to Seurat (v.3.2.0) [25], filtered (cells expressing > 500 detected genes, genes expressed in > 3 cells) and gene expression values were log normalized and scaled, then analyzed by principal component analysis (PCA). The first 15 principal components (PCs) were chosen to construct a K-nearst neighbors (KNN) graph and refine the edge weights between any two cells. Then we used “RunTSNE” function with the resolution parameter set as 0.7 to cluster cells, which identified 20 clusters and these clusters were renamed by accepted well known marker genes. The top 10 differentially-expressed genes (marker genes) of each cell cluster were determined by log fold change > 0.25 using default Wilcoxon rank-sum test [26]. The raw count matrices with cluster subsets (e.g., without testicular somatic cells) were imported to Monocle (v 2.10.0) [27] and used for additional combined cluster info. Differentially expressed genes or significantly variable genes among cells were identified and used for dynamic trajectory analysis which ordered cells in “pseudotime analysis”. With the gene count matrix as input, applied the function of “reduceDimension” and “orderCells” were carried out to generate the cell trajectory based on pseudotime. Psesudotime DEG analysis was performed using Monocle with a *P*-value cutoff of < 0.01. All heatmaps were generated in pseudotime order, and line plots were plotted in pseudotime order with fitted curves in the method of “auto” by ggplot2 package [28]. For further analysis, we isolated spermatogonia, differentiating spermatogonia and leptotene/Zygotene spermatocyte cells and performed these steps again; we obtained 10 clusters and renamed by accepted well known marker genes. Seurat was then used to perform differential expression analysis of merged matrices based on the non-parametric Wilcoxon rank-sum test.

### RNA-seq analysis

Total RNA was isolated and purified using Trizol reagent (Invitrogen) following the manufacturer’s procedure. The RNA quantities were measured using NanoDrop ND-1000 (Thermo Fisher Scientific, Waltham, MA, USA). The RNA integrity was assessed by Aligent 2100 with RIN number > 7.0. RNA high throughput sequencing was performed by Cloud-Seq Biotech (Shanghai, China). Briefly, total RNA was used for removing rRNAs with NEBNext rRNA Depletion Kit (New England Biolabs) following the manufacturer’s instructions. RNA libraries were constructed by using NEBNext^®^ Ultra™ II Directional RNA Library Prep Kit (New England Biolabs) according to the manufacturer’s instructions. Libraries were controlled for quality and quantified using the BioAnalyzer 2100 system (Agilent Technologies, Palo Alto, CA, USA). Library sequencing was performed on an illumine Hiseq instrument with 150 bp paired end reads. Paired-end reads were harvested from Illumina Hiseq-4000 sequencer, and were quality controlled by Q30. After 3′ adaptor-trimming and low qualities reads removing by cutadapt software (v1.9.3), the high qualities clean reads were aligned to the reference genome (Sscrofa11.1) with hisat2 software (v2.0.4). Then, guided by the Ensembl gtf gene annotation file, HTSeq software (v0.9.1) was used to get the raw count, and edgeR was used to perform normalization [29]. The differentially expressed mRNAs were identified by *P*-value and fold change. Enrichment analysis was performed based on the differentially expressed genes.

### Functional enrichment analysis with Metascape

We performed enrichment analysis of marker genes of each cell type in Metascape (http://metascape.org). For the given gene list, we converted these genes to human homology and performed with Metascape database based on human. The following ontology sources were chosen: GO Biological Processes, KEGG Pathway, Reactome Gene Sets and CORUM. All human genes had been used as the enrichment background. Cutoffs for significantly enriched terms were *P* < 0.01, minimum count of 3 and an enrichment factor > 1.5. The terms were grouped into clusters based on their membership similarities.

### Immunofluorescence assay

The isolated spermatogonia, pachytene spermatocytes and round spermatids were fixed with 4% paraformaldehyde for 25 min at 4 °C and washed with PBS for three times. Then, the cells were permeabilized for 10 min using 0.1% Triton-X 100 (Sigma-Aldrich) followed by washing with PBS for three time. The cells were further blocked with 10% donkey serum for 2 h at room temperature, and incubated with primary antibodies, including UCHL1 (Abcam, Catalog No. ab8189), SYCP3 (Abcam, Catalog No. ab15093) and CD63 (Proteintech, Catalog No. 25682–1-AP) at a dilution with 1:400 overnight at 4 °C. On the next day, the cells were washed with PBS for four times and incubated with secondary antibody (Yeason) at a dilution with 1:400 for 1 h at room temperature. The nuclei were labelled with DAPI (Bioworld Technology, St. Louis Park, MN, USA). A fluorescence microscope (Tokyo) was used for fluorescence observation and photographing.

The testis tissue was fixed in Bouin’s solution for 16 h, embedded in paraffin and sectioned. Sections were deparaffinized, rehydrated and heat-mediated antigen retrieval in 10 mmol/L sodium citrate buffer solution. After treatment with PBS for four times in 10 min, individual sections were incubated overnight at 4 °C. The following primary antibodies were used: CD99 (Proteintech, Catalog No.23079–1-AP), PODXL2 (R&D Systems, Catalog No.AF3534), KIT (Cell Signaling technology, Catalog No.3074S), UCHL1 (Abcam, Catalog No. ab8189), SYCP3 (Abcam, Catalog No. ab15093). Antigen reaction was conducted using with secondary antibody (Yeason) for 2 h at 4 °C in the dark. Then, the sections were incubated with DAPI (Bioworld Technology) to facilitate nuclear visualization. Images were obtained with Nikon Eclipse 80i fluorescence microscope camera (Tokyo).

#### Ethical statements

All experimental procedures involving animals were approved by the Northwest A&F University’s Institutional Animal Care and Use Committee.

### Data availability

The datasets generated in the current study are available in the NCBI GEO database under the accession number GSE174782.

## Results

### Cell partitioning through the analysis of porcine single cell transcriptomes

**Fig. 1:**
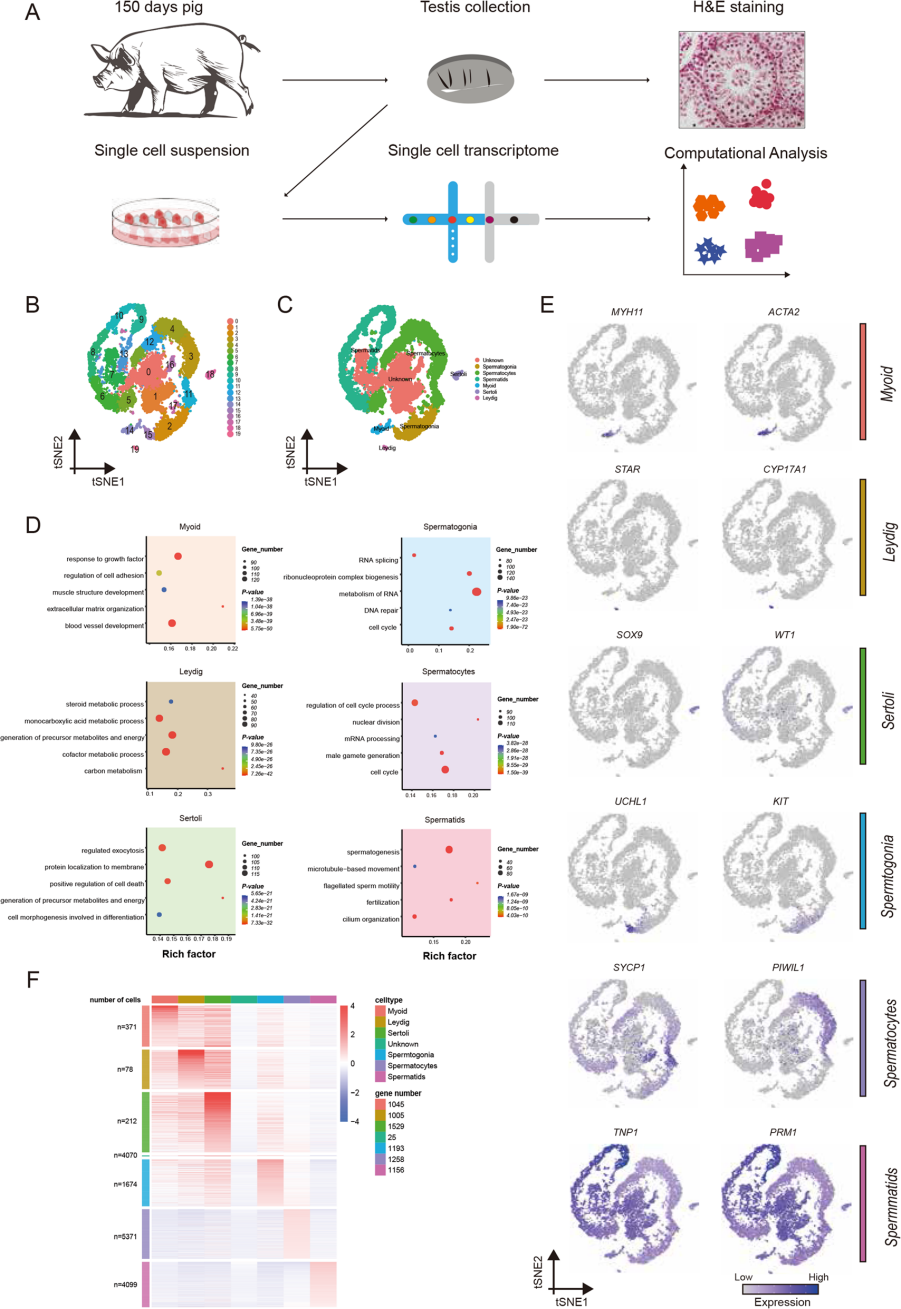
Single-cell transcriptome profiling and the cluster identification of porcine testicular cells. (**A**) Overview of the experimental design yielding droplet-based scRNA-Seq from porcine testis while using the contralateral testis for matched histology. (**B**) t-SNE plots show the 10 × Genomics profiling of unselected spermatogenic cells from porcine testis. Unbiased cell clusters are distinguished by color according to the key. (**C**) t-SNE plots show the identified cell types (cells are colored by the 6 broad cell types, and unknown cell type). (**D**) Enrichment terms and *P* values for the identified cell types (terms are colored by the 6 broad cell types). (**E**) Visualization of selected marker gene expression across all single cells in the t-SNE plot. For each cell cluster, 2 cell markers are shown in the Fig. (**F**) Scaled, normalized expression counts of the marker genes per cell type. Column and Row labels represent the cell type and the number of genes

Previous research on gene expression during spermatogenesis has relied largely on RNA-seq analysis of bulk cell populations. However, the heterogeneity in each population remains elusive. To uncover the heterogeneity, we dissociated porcine testis tissue from a 150-day-old Guanzhong black pig into single cells and performed 10 × Genomics scRNA-seq (Fig. 1A). We isolated single cells using a standard two-step enzymatic digestion procedure and removed the erythrocytes. The viability of the cells was about 95%. The cells were then used to construct the single-cell library. From a total of ~ 16,966 cells, 16,791 (99.97%) passed the standard quality control (QC) dataset filters and were retained for downstream analysis. The 842 Mb reads were obtained from the libraries, and the sequencing saturation rate was 30.40%. We obtained ~ 49.62 K reads/cell, and each cell had 19,690 unique molecular identifiers (UMIs) and 2759 genes detected (supplementary Table1). The cell-level quality metrics for the data were computed and shown in Fig. S1A and Fig. S1B.

To overcome the extensive technical noise in single genes of scRNA-seq data, we performed principal component analysis (PCA) on the matrix of scRNA-seq expression and selected the top 15 components for subsequent analysis. Cell partitioning via t-distributed stochastic neighbor embedding (t-SNE) analysis identified 20 clusters (Fig. 1B), and the number of the cells in each cluster was shown in supplementary Table1. As the markers of porcine male germ cells were not fully available, we drew on the markers in humans. The porcine protein coding genes were mapped to their human 1:1 orthologs. Based on these markers, 20 clusters were defined as 6 broad cell types, while other clusters were classified as unknown cell types (Fig. 1C). Specifically, germ cells and somatic cells could first be distinguished by the mutually exclusive expression of two genes, *DDX4* in germ cells and *VIM* in somatic cells as previously reported [19]. The germ cells with *DDX4* expression were further sub-classified as follows: spermatogonia (*UCHL1*, *ZBTB16*, *KIT*, *STRA8* and *DMRT1*), spermatocytes (*DMC1*, *RAD51AP2*, *HORAD1*, *SYCP1*, *PIWIL1* and *NME8*) and spermatids (*FHL5*, *TPPP2*, *PRM1* and *TNP1*). Somatic cells were identified: Sertoli cells (*GATA4*, *SOX9*, *AR* and *WT1*), Leydig cells (*CYP11A1*, *CYP17A1* and *STAR*), and myoid cells (*MYH11* and *ACTA2*) (Fig. 1E, Fig. S1D).

The marker genes were calculated by Seurat, which revealed the underlying features of gene expression in each cell type. The numbers of cells and genes in each cell type were shown in Fig. 1F. Dynamic changes in expression of the marker genes were presented Fig. S1C, in accordance with the previous reports in humans [17, 18]. To identify the functional categories associated with each cell type, we utilized the Metascape (http://metascape.org) for functional enrichment analysis, which provided further insights into the functional theme and regulatory network in porcine somatic cells and germ cells. For instance, genes in spermatogonia were enriched in cell cycle, DNA repair and transcriptional regulation by *TP53*, indicating the active proliferation of spermatogonia. The genes in spermatocytes were involved in meiotic cell cycle and regulation of chromosome organization. The genes in spermatids represented more downstream processes, such as spermatogenesis, fertilization and cilium organization (Fig. 1D). As to the testicular somatic cells, genes in myoid cells were enriched with response to growth factor, extracellular matrix organization and regulation of cell adhesion. The genes in Leydig cells were involved in monocarboxylic acid metabolic process and steroid metabolic process, consistent with the main function of Leydig cells for testosterone synthesis. The genes in Sertoli cells were related to regulation of cell death and exocytosis. Thus, the enrichment analysis enables more knowledge about the potential function of these major cell types.

### Isolation and assay of porcine spermatogonia, spermatocytes and spermatids

**Fig. 2:**
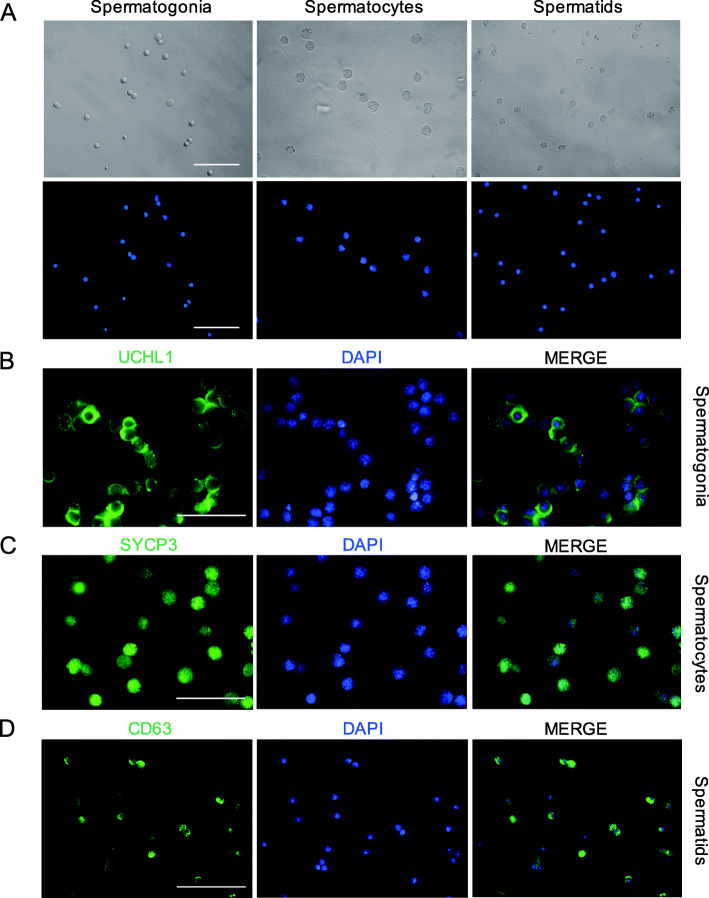
Identification of porcine male germ cells. (**A**) The morphology of the freshly isolated porcine spermatogonia, spermatocytes and spermatids. Scale bar = 50 μm. (**B**) Immunocytochemistry staining for UCHL1 in spermatogonia. (**C**) Identification of SYCP3 the freshly isolated spermatocytes. (**D**) CD63 was identified to express in the spermatids. Scale bar = 50 μm

To link our computationally-defined clusters to morphologically-defined germ cell types, we collected porcine spermatogonia, spermatocytes and spermatids using STA-PUT, a velocity sedimentation approach. Different cell types can be distinguished based on the cell size and nuclear morphology. Spermatogonia are 10 μm in diameter and have ovoid nuclei, and spermatocytes are about 15 μm in diameter with patchy condensed chromatin and large nuclei, while spermatids are approximately 5 μm in diameter and have a round nucleus with a high nucleus-to-cytoplasm ratio and highly condensed DNA (Fig. 2A). The viability of the freshly isolated porcine spermatogonia, spermatocytes and spermatids were over 95%.

Subsequently, immunofluorescence staining was performed to detect the cell purity. UCHL1 was selected as the marker of spermatogonia (Fig. 2B). SYCP3, the axial/lateral elements of synaptonemal complex proteins, was selected as the marker of spermatocytes (Fig. 2C). Interestingly, we found that CD63, a cell surface protein, could be used as a new marker for porcine spermatids, as confirmed by immunofluorescence and immunohistochemistry analyses (Fig. S2A, Fig. S2B). Consequently, CD63 was chosen to identify the purity of the collected spermatids (Fig. 2D). Overall, the purity of isolated germ cells was more than 80%, suggesting that spermatogonia, spermatocytes and spermatids were successfully enriched.

### Verification of porcine germ cell types by bulk RNA-seq

Upon successful enrichment of spermatogenic populations, we performed bulk RNA-seq to validate the scRNA-seq result, which could exhibit the similar gene expression and GO results between scRNA-seq and bulk RNA-seq in spermatogonia, spermatocytes and spermatids. For bulk RNA-seq analysis, we constructed transcriptomic libraries from 3 biological replicates per cell type. We calculated the Pearson correlation coefficient between the samples and the annotated cell types in scRNA-seq dataset, and one sample of spermatocytes was excluded due to the low repeatability (Fig. S3B).

**Fig. 3:**
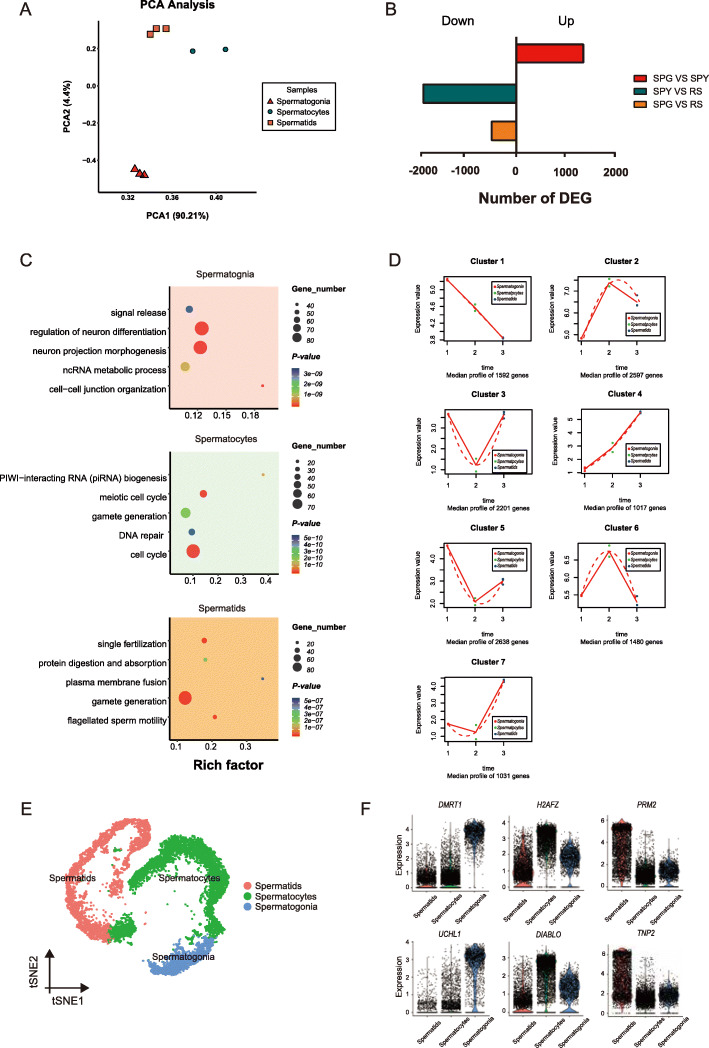
Comparative analysis of the data between bulk RNA-seq and scRNA-seq. (**A**) Results of principal components analysis (PCA) for the bulk RNA-seq samples, spermatogonia, spermatocytes and spermatids. (**B**) Differentially expressed genes (DEGs) for each pairwise comparison. (**C**) Representative GO terms and *P* values are shown for each cell type. (**D**) Data visualization according to the cluster analysis. Each plot shows the average expression profile of the gene clusters from all samples. Dots show the actual average expression values for each sample (red represent spermatogonia, green represent spermatocytes and the blue represent spermatids). (**E**) t-SNE plots show the identified cell types (cells are colored by the germ cell types). (**F**) Visualization of marker gene expression across germ cells in the violin plot

The reproducibility for other samples was validated by PCA analysis (Fig. 3A). The total number of expressed genes exhibited dynamic changes during porcine spermatogenesis, with 12,974 expressed in spermatogonia, 11,456 in spermatocytes, and 13,220 in spermatids. The abundance of protein coding genes exhibited considerable variations in each cell type (Fig. S3A). Pairwise differential gene expression analysis between cell types generated lists of differentially expressed genes (DEGs; fold change > 2, FDR < 0.05). A total of 9919 DEGs were identified between spermatogonia and spermatocytes, of which 5626 were upregulated and 4293 were downregulated. From spermatocytes to spermatids, there were 2799 upregulated genes and 4742 downregulated genes. From spermatogonia to spermatids, 3680 were upregulated and 4126 were downregulated. Consistent with previous reports in mice [30], gene expression of the protein coding genes had global shutdown during meiosis, and the pairwise comparisons were more negative in spermatocytes, suggesting the lower transcriptional activity in spermatocytes (Fig. 3B).

To explore the possible functions of stage-specific genes, maSigPro was used to perform time series analysis [31]. The analysis revealed that 12,556 variable genes were clustered into seven groups (Fig. S3C). Among them, the trends of gene expression manifested that the genes in cluster 1 were specifically expressed in spermatogonia, while cluster 6 and cluster 7 matched spermatocytes and spermatids, respectively (Fig. 3D). In addition, there were 1592, 1480, and 1031 stage-specific genes in spermatogonia, spermatocytes, and spermatids, respectively. Subsequently, we performed functional enrichment analysis for the genes in these clusters. The upregulated genes in spermatogonia were enriched with ncRNA metabolic process. The 1480 upregulated genes in spermatocytes were involved in meiotic cell cycle, PIWI-interacting RNA (piRNA) biogenesis and gamete generation. Finally, Metascape analysis revealed that the upregulated genes in spermatids were enriched with single fertilization and flagellated sperm motility (Fig. 3C). Taken together, the functional enrichment results showed the similarity of the identified cell types by scRNA-seq to those by bulk RNA-seq. In addition, we clustered porcine germ cells in scRNA-seq (Fig. 3E), and matched the bulk and scRNA-seq data by using classification of the bulk samples based on stage-specific marker genes obtained from the defined cell types in scRNA-seq data (Fig. 3F). The Pearson correlation results further verified our correct classification (Fig. S3B). Thus, by comparing bulk RNA-seq data to those of scRNA-seq, we validated the accuracy of the defined porcine germ cell types.

### Cellular heterogeneity during porcine spermatogonial differentiation

**Fig. 4:**
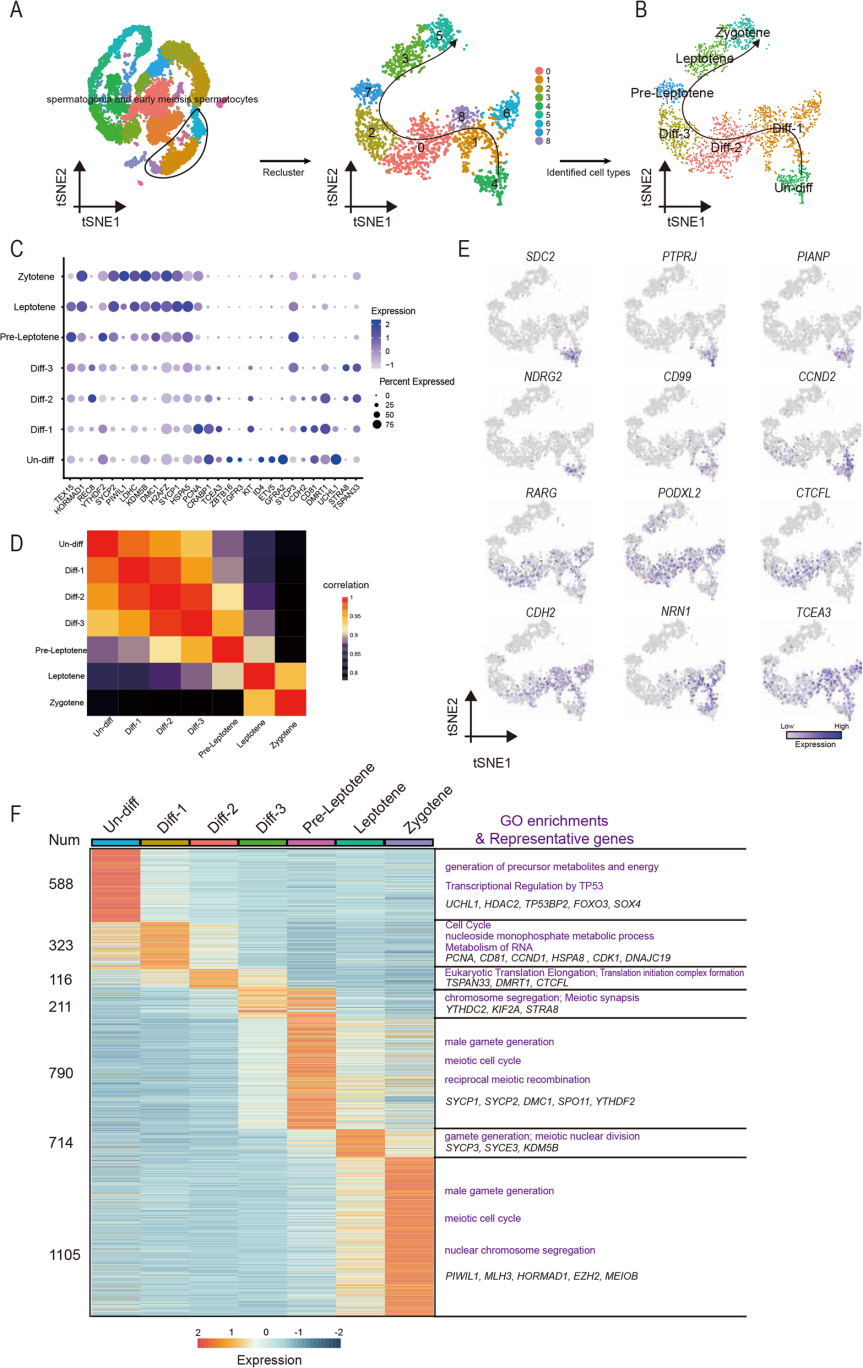
Single-cell porcine spermatogonia trajectories reveal transitions with differentiation and initiation of meiosis. (**A**) Focused analysis (t-SNE and clustering) of the mitosis and initiation of meiotic germ cells reveals differentiation progression of spermatogonia. (**B**) t-SNE plot reveal the states in spermatogonia differentiation. (**C**) Dot plot depicts the expression of marker genes in each state. The dot size represents the percentage of cells expressed and the color intensity represents relative expression level. (**D**) The correlation across each state and illustrated by Pearson correlation analysis. (**E**) Expression patterns of new defined marker genes for undifferentiated and differentiating spermatogonia visualized in t-SNE plots. (**F**) Heatmap of marker genes from each state. Top, cell types; Left, number of marker genes; Right, representative GO terms and representative genes

To capture the fate transition in porcine germ cells, we re-clustered the clusters 15, 2, 17 and 11 that represented spermatogonia and early meiotic spermatocytes to analyze the subgroups of spermatogoinal differentiation and meiotic entry. Cells were ordered via the pseudo-time method, and the analysis determined trajectories of differentiation according to the marker gene expression patterns. The pseudo-time analysis provided an arrow vector which aligned with the developmental order of spermatogonia (Fig. S4A). The top 50 variable genes across pseudo-time were shown in Fig. S4B. Next, we performed re-clustering of these cell types, which revealed nine sub-clusters (Fig. 4A). Based on the known markers (Fig. S4C), we detected one cluster (Un-diff) that pertained to undifferentiated spermatogonia based on the expression of *UCHL1* and a small number of cells that expressed SSC markers (*ZBTB16*, *ETV5*, *ID4* and *FGFR3*) [32–34]. Moreover, based on expression of *CD81*, *PCNA*, *KIT*, *STRA8*, *TSPAN33* and *DMRT1*, we assigned five clusters to three stages of differentiating spermatogonia (Diff-1, Diff-2 and Diff-3) [18, 35–37]. The expression of *PCNA* and *DMRT1* suggests that the differentiating spermatogonia are highly proliferative, which mediates the transition of mitosis to meiosis [38, 39]. Besides, marker genes involved in the synaptonemal complex (*SYCP1*, *SYCP2* and *SYCP3*) and meiotic recombination (*DMC1*) identified three clusters as pre-leptotene spermatocytes (Pre-Leptotene), leptotene spermatocytes (Leptotene) and zygotene spermatocytes (Zygotene) [40]. Thus, we obtained cell-type identities for spermatogonial subpopulations and revealed the early stages of meiosis (Fig. 4B). The expression of marker genes in each cell types was shown in Fig. 4C. In addition, we analyzed the correlation of the identified cell types by Pearson analysis, and found low correlation between mitosis and meiosis (Fig. 4D).

Next, we selected marker genes in each cluster and the dynamic changes of gene expression were shown in Fig. 4F. To investigate the different functions of these clusters, functional enrichment analysis of marker genes was performed (Fig. 4F). The marker genes in Un-diff were significantly enriched with generation of precursor metabolites and energy and transcriptional regulation by *TP53*. Diff-1 genes-enriched terms included cell cycle, nucleoside monophosphate metabolic processand metabolism of RNA, consistent with the notion that differentiating spermatogonia are undergoing proliferative expansion [41]. Significantly enriched terms for Diff-2 genes included eukaryotic translation elongation and translation initiation complex formation. Remarkably, the marker genes in Diff-3 were involved in meiotic synapsis and chromosome segregation, which suggested that the genes at this stage point to subsequent entry into meiosis. In addition, the marker genes in Pre-Leptotene, Leptotene and Zygotene were significantly enriched with meiotic process and gamete development. Together, these results revealed that many biological processes are active in porcine spermatogonial subsets.

To further explore cell-type specific genes for porcine undifferentiated and differentiating spermatogonia, we filtered a series of marker genes in each cluster. We analyzed the dynamic expression changes of these genes during spermatogonial differentiation (Fig. 4E). The analysis indicated that *SDC2*, *PTPRJ*, *PIANP*, *NDRG2*,* CD99* and *CCND2* were specifically expressed in Un-Diff, while *RARG*, *PODXL2*, *CTCFL* and *CDH2* were highly expressed in Diff-1 ~ 3. In addition, *NRN1* and *TCEA3* were highly expressed in both Un-Diff and Diff-1 ~ 3. Remarkably, these genes were specifically expressed in spermatogonia (Fig. S4D). Thus, our analysis confirmed many known genes and provided a list of key candidate genes with undefined functions in porcine spermatogonial development, thereby facilitating future studies in this respect.

### Identification of cellular localization for CD99 and PODXL2 in porcine testes

**Fig. 5:**
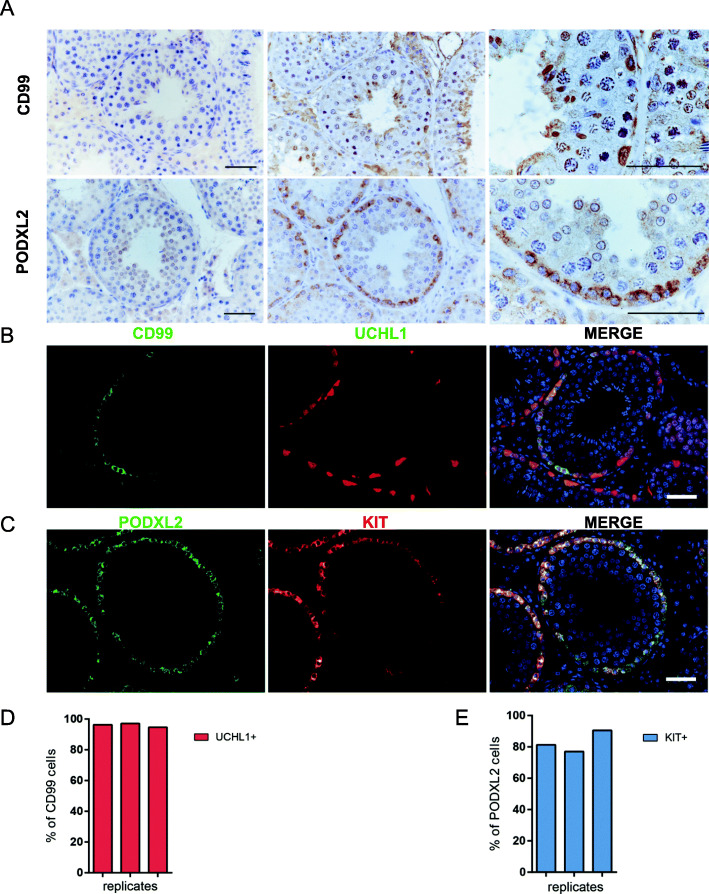
Expression of CD99 and PODXL2 in porcine testis. (**A**) Immunohistochemical staining of CD99 and PODXL2 in porcine testis. Scale bars = 50 μm. Coimmunofluorescence examination of the expression of (**B**) CD99 with UCHL1 and (**C**) PODXL2 with c-KIT in porcine testis. Scale bars = 50 μm. Data are normalized by the number of double-positive cells for three independent experiments (**D**) CD99 with UCHL1 and (**E**) PODXL2 with c-KIT

To identify the novel markers from the candidate genes (Fig. 4E), we performed immunohistochemistry staining for CD99 and PODXL2. Immunohistochemical analysis revealed that CD99 and PODXL2 localized in spermatogonia adjacent to the basement membrane of seminiferous tubules (Fig. 5A). According to the single-cell RNA-seq data, CD99 and PODXL2 were expressed in different subpopulations of porcine spermatogonia. Of this, CD99 was primarily enriched in undifferentiated spermatogonia, while PODXL2 is preferentially expressed in differentiating spermatogonia. To validate this, we performed double immunofluorescence staining for CD99/UCHL1 and PODXL2/KIT (Fig. 5B, C). The result showed that CD99 staining partly overlapped with that of UCHL1, an undifferentiated spermatogonial marker (96.03% ± 1.02%), indicating that CD99^+^ cells represent a subset of undifferentiated spermatogonia (Fig. 5D). The colocalization analysis for PODXL2 and KIT showed that the majority of PODXL2^+^ cells were also positive for KIT, a differentiating spermatogonial marker (82.94% ± 5.66%), suggesting that PODXL2 marks the differentiating spermatogonial population in porcine testes (Fig. 5E). Taken together, our result revealed that CD99 and PODXL2 could serve as reliable markers for porcine spermatogonial subpopulations.

## Discussion

Mammalian spermatogenesis is a complicated and dynamic cellular differentiation process. Gene expression profiles of male germ cells in mice and humans have been reported previously [16, 18, 42]. However, the information in pigs remains largely unclear. In this study, we used scRNA-seq and performed a comprehensive analysis of gene expression during porcine spermatogenesis. Our analysis of ~ 16,966 individual testicular cells from the adult porcine testis afforded the maximum resolution of gene expression patterns associated with the full collection of cells within the spermatogenic cell lineage. We identified the subtypes of male germ cells and testicular somatic cells. Our single-cell data corroborate that spermatogenesis represents a continual process of ever-changing gene expression profiles.

### Catalog of germ cells and somatic cells

Based on the germ cell marker *DDX4* and somatic cell marker *VIM* [19], the testicular cells could be first separated. Overall, we identified all major male germ cell types in porcine testes, including spermatogonia, spermatocytes and spermatids. The dynamic changes in expression of the marker genes were consistent with the previous reports in humans [17, 18]. In addition, we identified Sertoli cells, Leydig cells and myoid cells (Fig. 1E, Fig. S1D). The marker genes in myoid cells were enriched with response to growth factor, extracellular matrix organization and regulation of cell adhesion. The genes in Leydig cells were involved in synthesis for testosterone synthesis [43]. In Sertoli cells, the marker genes were involved in regulation of cell death and exocytosis.

In the present study, we identified spermatogonia, spermatocytes, spermatids, Sertoli cells, Leydig cells and myoid cells through scRNA-seq. Recently, Yang et al. reported characterization of sheep spermatogenesis via scRNA-seq [20]. However, spermatogonia cluster was not identified in their study. Here, by comparing scRNA-seq and bulk RNA-seq data, we further validated the accuracy of the defined porcine germ cell types.

### Spermatogonial subsets

Our scRNA-seq analysis revealed the presence of 4 spermatogonial subsets: Un-diff, Diff-1, Diff-2, and Diff-3 SPG (Fig. 4B). Un-diff SPG matched undifferentiated spermatogonia based on expression of *UCHL1, ZBTB16, ETV5, ID4* and *FGFR3* [32–34]. In Diff-1 subset, compared to Un-diff, UCHL1 expression decreased and expression of *KIT* (a marker for differentiating SPG) and *PCNA* (a marker for cell proliferation) increased. Diff-1 spermatogonia were enriched with genes involved in cell cycle, nucleoside monophosphate metabolic process and metabolism of RNA, which is consistent with the notion that differentiating spermatogonia are undergoing proliferative expansion [41]. Thus, Diff-1 subset may correspond to type A1-A4 spermatogonia [4, 44]. In Diff-2 subset, *KIT* expression remained at a high level, and *DMRT1* expression decreased slightly, while *STRA8* expression initiated (Fig. 4C, supplementary Table2). DMRT1 promotes spermatogonial differentiation and controls the mitosis/meiosis switch [45, 46]. In mouse spermatogonia, DMRT1 directly represses STRA8 transcription and activates spermatogonial differentiation [47]. In Diff-3 subset, *DMRT1* expression decreased sharply, while *STRA8* expression further increased, and SYCP2/3 essential for the assembly of the synaptonemal complex was upregulated, in agreement with that in humans [18]. As *DMRT1* and *STRA8* are reciprocally expressed in B-spermatogonia and preleptotene spermatocytes [45], the Diff-2 subset is likely to represent intermediate and early type B spermatogonia and Diff-3 subset may represent type B spermatogonia at the late stage and ready for transition to preleptotene spermatocytes. Thus, this study has for the first time systematically investigated the spermatogonial subtypes in livestock. Our refined assignments of spermatogonial subtypes will facilitate future research on the mechanisms regulating the balance between SSC self-renewal and differentiation.

### Spermatogonial markers

In the present study, the scRNA-seq analysis revealed twelve candidate spermatogonial markers. We explored the localization of CD99 and PODXL2 in porcine testes and found that they were localized in spermatogonia at the periphery of the seminiferous tubules (Fig. 5A). Double immunofluorescence staining for CD99/UCHL1 revealed that 96.03 of CD99^+^SPG overlapped with UCHL1^+^ cells (Fig. 5D). CD99 is a cell surface glycoprotein that functions as a cell-cell adhesion factor [48]. Previous studies indicate that CD99 is a marker of stem cells in acute myeloid leukemia (AML) and myelodysplastic syndrome, and is also a marker for prospective separation of leukemic stem cells (LSCs) from functionally normal hematopoietic stem cells in AML [49]. Intriguingly, CD99 has been reported to be expressed in testes [50]. As CD99 colocalized with UCHL1, CD99^+^ cells could represent a subset of undifferentiated spermatogonia in pigs. It would be interesting to study the role of CD99 during spermatogonial proliferation and differentiation in future studies.

PODXL2 is a transmembrane type I sialomucin protein [51]. It promotes epithelial-to-mesenchymal (EMT) transition by disrupting cell-cell junctions [52, 53].PODXL2 depletion led to reduction of OCT4 and Nanog expression and affected cell proliferation in BT474 cells [54]. Here, we demonstrated that the majority of PODXL2^+^ cells were also KIT^+^, suggesting that PODXL2 marks the differentiating spermatogonial population in porcine testes. KIT is a well-established marker that can be used for isolation and enrichment of differentiating spermatogonia via fluorescence-activated cell sorting in mice and humans [55, 56]. However, in our preliminary study, several commercial c-kit antibodies could not be used for sorting porcine early germ cells. Thus, PODXL2, as a novel marker for differentiating spermatogonia, may be conducive to characterization of spermatogonial differentiation in pigs, and probably in other farm animals as well, which warrants future investigation. Future studies can also be carried out to explore the role of PODXL2 in regulation of spermatogonial differentiation.

## Conclusions

We identified four subsets of spermatogonia, spermatocytes, spermatids and three somatic cell types in porcine testes through scRNA-seq. Our scRNA-seq revealed twelve candidate spermatogonial markers, and two novel cell surface markers for porcine spermatogonia were validated, which would be conducive to characterization of spermatogonial differentiation in pigs. Collectively, our datasets offer valuable information on porcine spermatogenesis, and pave the way for identification of key molecular markers involved in development of male germ cells.

## Supplementary Information

**Additional file 1 supplementary Table1**. List of data summary and the number of cells in each cluster.

**Additional file 2 supplementary Table2**. The expression of the genes involved in spermatogonia proliferation and differentiation in spermatogonia subsets

**Additional file 3 Fig. S1 Data quality and cell type annotation by other genes (*Relates to*** Fig. 1**).** (**A**) Distribution of cell attributes in the pig dataset. (**B**) The correlation between the number of genes and UMI counts. (**C**) Heatmap showed expression of 45 marker genes in 6 cell types. (**D**) Visualization of marker gene expression across unselected clusters in the violin plot. Unbiased cell clusters are distinguished by color according to the key.

**Additional file 4 Fig. S2 Expression of CD63 in porcine testis.** (**A**) Coimmunofluorescence examination of the expression of *CD63* with PNA. Scale bars = 50 μm. (**B**) Immunohistochemical staining of CD63 in porcine testis. Scale bars = 50 μm.

**Additional file 5 Fig. S3 (*Relates to*** Fig. 3**)** (**A**) Density plot show the distribution of gene expression in spermatogonia, spermatocytes and spermatids. (**B**) The correlation between bulk and scRNA-seq data illustrated by Pearson correlation analysis. (**C**) Data visualization according to the cluster analysis. Each line shows a single gene expression from all samples.

**Additional file 6 Fig. S4 (*Relates to*** Fig. 4**)** (**A**) Single-cell transcriptomes from porcine undifferentiated spermatogonia, differentiating spermatogonia, Leptotene and Zygotene spermatocytes were used for cell trajectories ordered in pseudotime (right) and cells colored according to cell cluster (left). (**B**) Heatmaps show the top 50 variable genes across pseudotime from porcine undifferentiated spermatogonia, differentiating spermatogonia, Leptotene and Zygotene spermatocytes (scaled expression according to legend). (**C**) Visualization of marker gene expression across the differentiation states of spermatogonia in the violin plot. Unbiased cell clusters are distinguished by color according to the key. (**D**) Expression patterns of new defined marker genes for undifferentiated and differentiating spermatogonia visualized in t-SNE plots for all cell types.

## Data Availability

All data supporting our findings are included in the manuscript.

## Abbreviations

AML: Acute myeloid leukemia
BSA: Bovine serum albumin
DEGs: Differentially expressed genes
FACS: Fluorescence-activated cell sorting
GEMs: Gel bead emulsions
KNN: K-nearst neighbors
Leptotene: Leptotene spermatocytes
LSCs: Leukemic stem cells
MACS: Magnetic-activated cell sorting
PCA: Principal component analysis
Pre-Leptotene: Pre-leptotene spermatocytes
QC: Quality control
RNA-seq: RNA-sequencing
scRNA-seq: Single-cell RNA sequence
SSCs: Spermatogonial stem cells
t-SNE: T-distributed stochastic neighbor embedding
Zygotene: Zygotene spermatocytes
