# Supplementary figures and images for: Single-cell RNA-sequencing reveals the dynamic process and novel markers in porcine spermatogenesis
Source: J Anim Sci Biotechnol. 2021 Dec 7;12:122. doi: 10.1186/s40104-021-00638-3 (PMC8650533; doi:10.1186/s40104-021-00638-3)

A

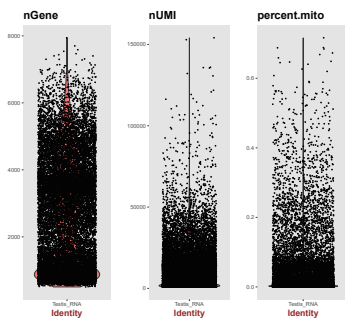

B

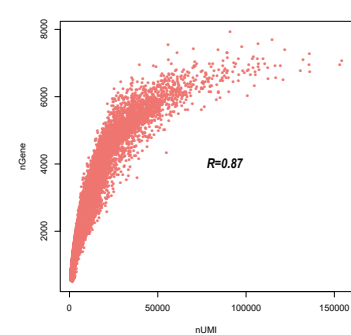

C

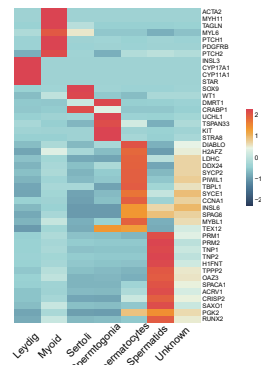

D

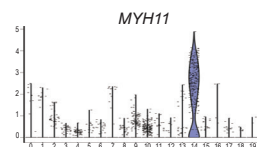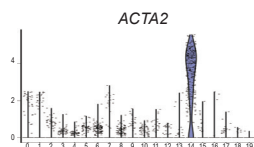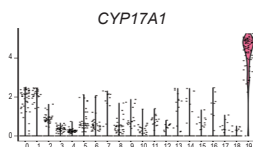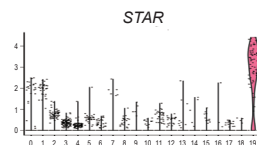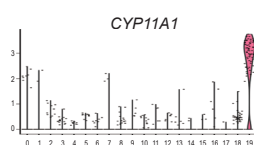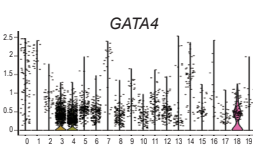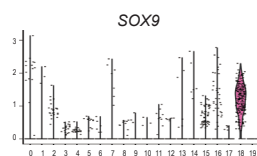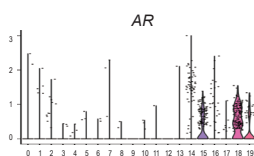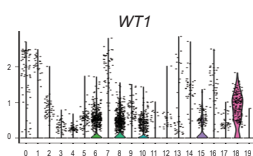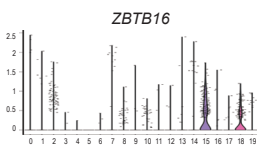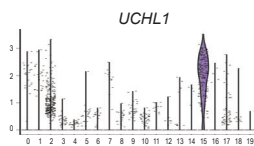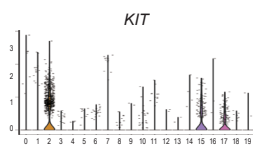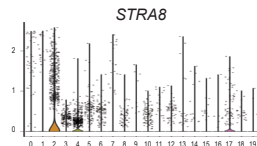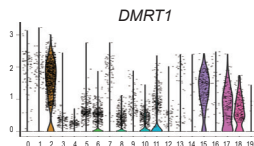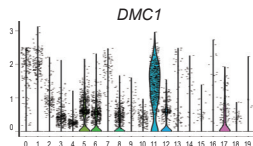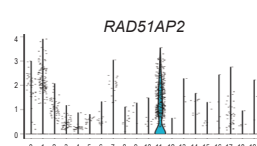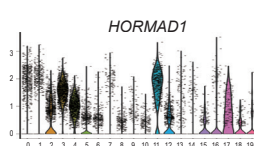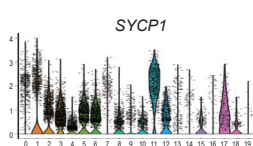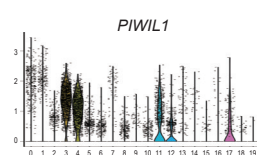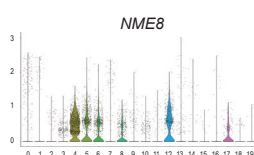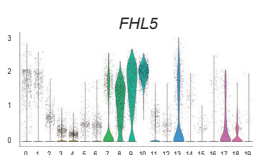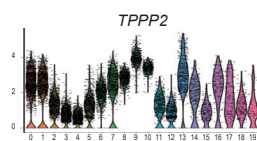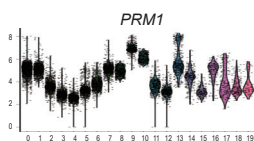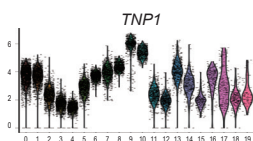

Supplement: Supplementary file 3 — Additional file 3 Fig. S1 Data quality and cell type annotation by other genes (Relates to Fig. 1). (A) Distribution of cell attributes in the pig dataset. (B) The correlation between the number of genes and UMI counts. (C) Heatmap showed expression of 45 marker genes in 6 cell types. (D) Visualization of marker gene expression across unselected clusters in the violin plot. Unbiased cell clusters are distinguished by color according to the key. [file 40104_2021_638_MOESM3_ESM.pdf]

A

PNA

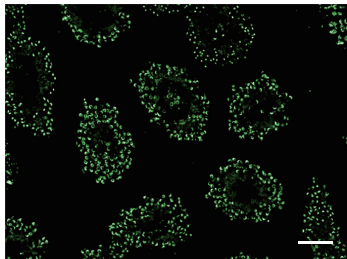

CD63

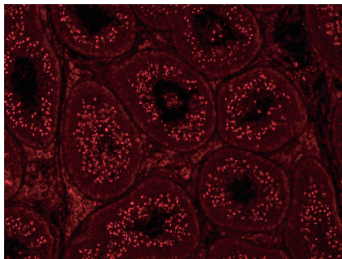

Merge

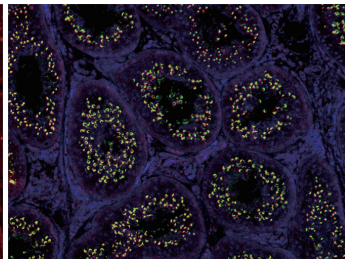

B

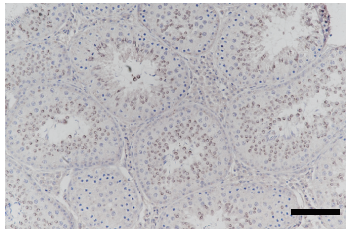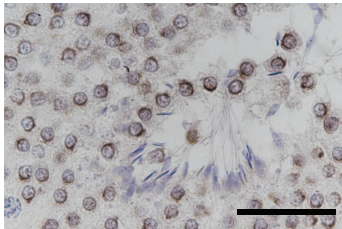

Supplement: Supplementary file 4 — Additional file 4 Fig. S2 Expression of CD63 in porcine testis. (A) Coimmunofluorescence examination of the expression of CD63 with PNA. Scale bars = 50 μm. (B) Immunohistochemical staining of CD63 in porcine testis. Scale bars = 50 μm. [file 40104_2021_638_MOESM4_ESM.pdf]

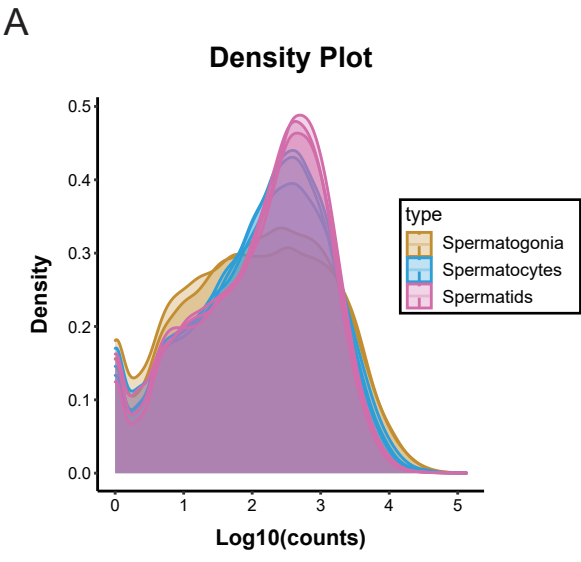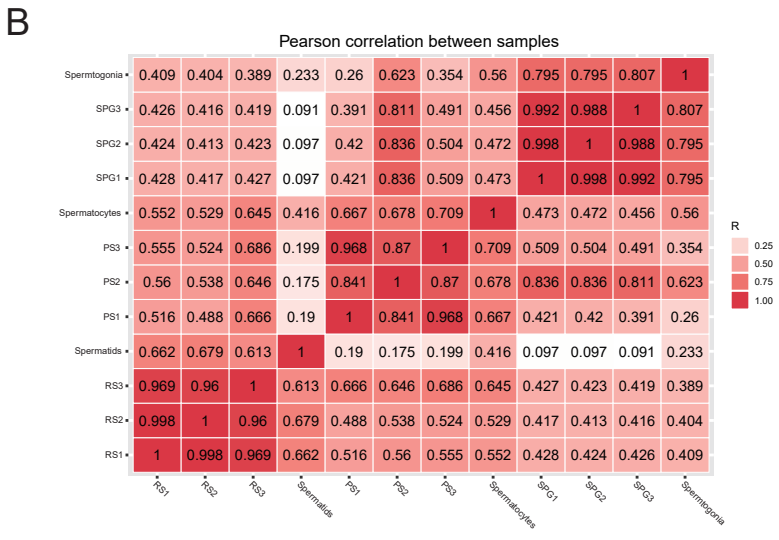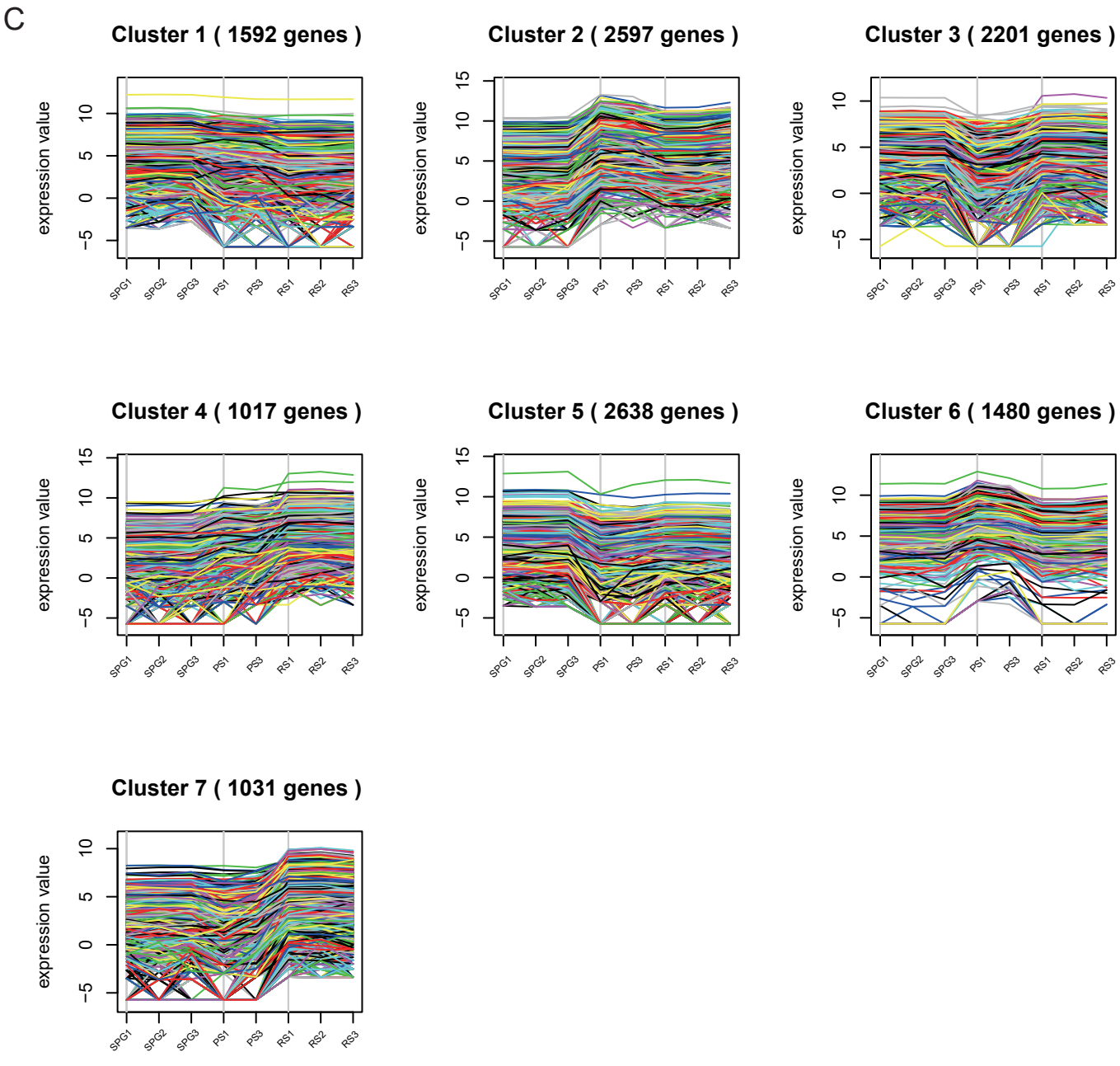

Supplement: Supplementary file 5 — Additional file 5 Fig. S3 (Relates to Fig. 3) (A) Density plot show the distribution of gene expression in spermatogonia, spermatocytes and spermatids. (B) The correlation between bulk and scRNA-seq data illustrated by Pearson correlation analysis. (C) Data visualization according to the cluster analysis. Each line shows a single gene expression from all samples. [file 40104_2021_638_MOESM5_ESM.pdf]

A

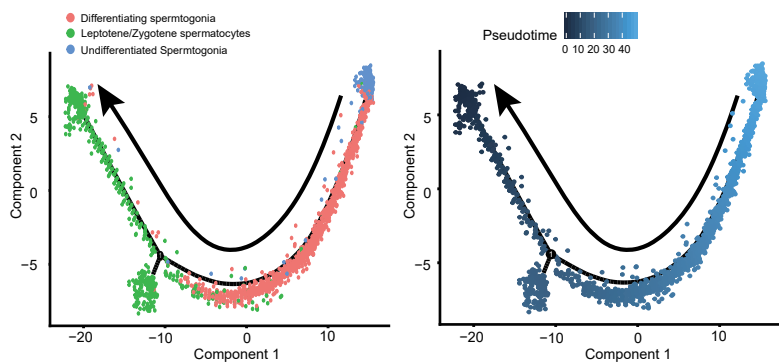

B

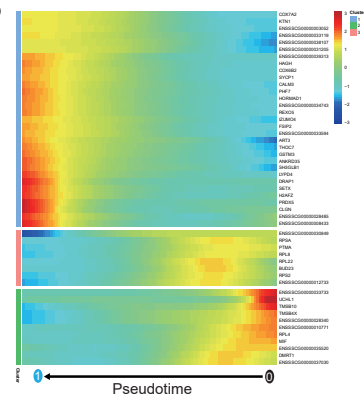

C

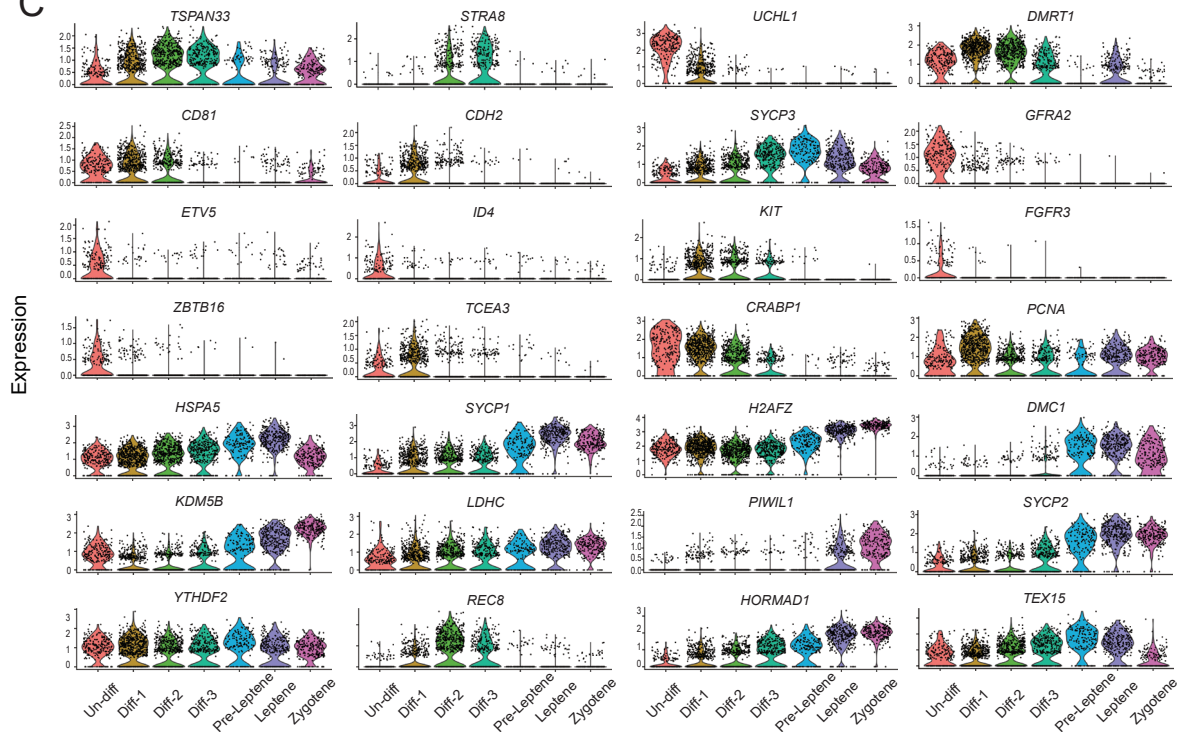

D

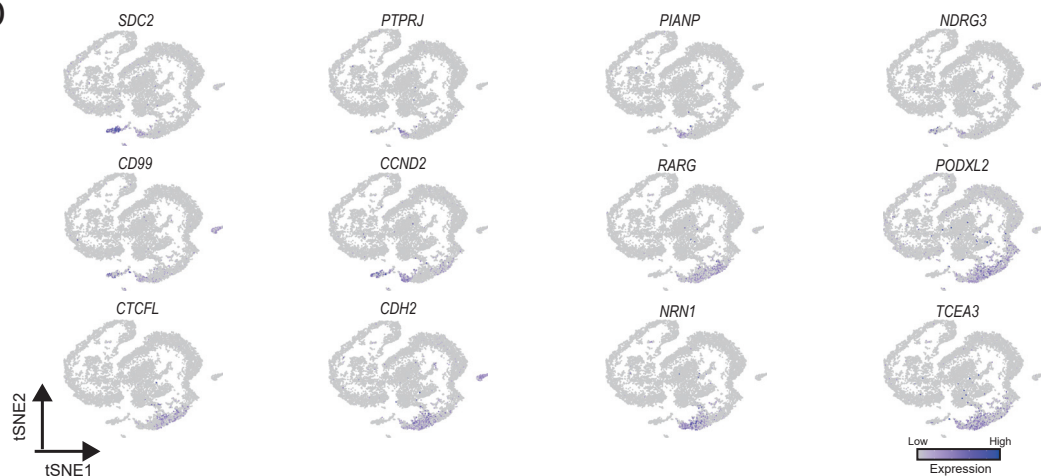

Supplement: Supplementary file 6 — Additional file 6 Fig. S4 (Relates to Fig. 4) (A) Single-cell transcriptomes from porcine undifferentiated spermatogonia, differentiating spermatogonia, Leptotene and Zygotene spermatocytes were used for cell trajectories ordered in pseudotime (right) and cells colored according to cell cluster (left). (B) Heatmaps show the top 50 variable genes across pseudotime from porcine undifferentiated spermatogonia, differentiating spermatogonia, Leptotene and Zygotene spermatocytes (scaled expression according to legend). (C) Visualization of marker gene expression across the differentiation states of spermatogonia in the violin plot. Unbiased cell clusters are distinguished by color according to the key. (D) Expression patterns of new defined marker genes for undifferentiated and differentiating spermatogonia visualized in t-SNE plots for all cell types. [file 40104_2021_638_MOESM6_ESM.pdf]
